# Cost-Effectiveness of Operative Versus Non-Operative Treatment for Clavicle Fracture: a Systematic Literature Review

**DOI:** 10.1007/s12178-020-09640-0

**Published:** 2020-05-08

**Authors:** Gilber Kask, Lauri Raittio, Ville M. Mattila, Antti P. Launonen

**Affiliations:** 1 Department of Orthopaedics and Traumatology, Unit of Musculoskeletal Surgery, Tampere University Hospital, Teiskontie 35, 33521 Tampere, Finland; 2 Faculty of Medicine and Life Sciences, Tampere University, Kalevantie 4, 33100 Tampere, Finland

**Keywords:** Clavicle, Cost-effectiveness, Fracture, Treatment

## Abstract

**Purpose of Review:**

Operative and non-operative treatment of midshaft clavicle fractures seems to yield comparative functional results. Furthermore, it has been suggested that surgery is more expensive compared with non-operative treatment of clavicle fracture. Cost-effectiveness seems to be more important in trends of treatment decisions. The purpose of this study is to investigate the cost-effectiveness of clavicle fracture treatment.

**Recent Findings:**

Seven publications were selected, and 5 studies showed that operative treatment is more expensive than non-operative treatment. The mean overall cost per person in discounted prices was 10,230 USD for operative and 7923 USD for non-operative treatment. The mean absence from work ranged 8–193 and 24–69 days for operative and non-operative treatment, respectively. Studies varied in methods of assessing the cost-effectiveness of treatment modalities.

**Summary:**

Based on this literature review, routine operative treatment seems to be more expensive. In some cases, operative treatment might be more cost-effective. In all studies, direct and indirect costs of health care were calculated, but a great heterogeneity exists in the sources of cost data between countries. The cost-effectiveness of the treatment of clavicle fracture depends strongly on the cost of operative treatment and length of absence from work. Cost-effectiveness analysis could be a routine in RCT studies in the future.

**Electronic supplementary material:**

The online version of this article (10.1007/s12178-020-09640-0) contains supplementary material, which is available to authorized users.

## Introduction

Clavicle fractures in the adult population represent approximately 3% of all fractures and 44% of those in the shoulder area [1]. The current care in clavicle fractures is either operative or non-operative treatment. Widely accepted indications for operative intervention in clavicle fractures include open fractures, fractures associated with skin compromise, and concomitant neurological or vascular injury. Relative indications for operative treatment include fractures with more than 2-cm shortening, severe displacement of the fracture, concomitant chest injuries, high-energy injuries, a floating shoulder, and fracture non-unions [2–8]. Operative implants and techniques vary, but open reduction with plate fixation is the most widely used operative technique for clavicular fixation [9, 10].

The incidence of operative treatment has increased during the last decade [11]. To the best of our knowledge, 9 RCTs comparing open reduction with plate fixation and non-operative treatment exist [8, 9, 12•, 13, 14, 15, 16, 17•, 18]. The results of these recent RCTs show that there is little or no difference in functional outcome at 1- and 2-year follow-up between operative and non-operative treatment. Operative treatment, however, may be an option for patients who need a quick recovery and for those who have risk factors for non-union, such as large displacement and comminution of fracture [19, 20]. On the other hand, operative intervention has a relatively high complication rate (≥ 23%) that includes infection, non-union, and implant failure [13, 21–23]. Furthermore, comparison between the different RCTs is demanding due to high heterogeneity of outcomes [24].

The increased costs of health care have also increased the importance of understanding and applying economic evaluations, including the cost-effectiveness of treatment methods, in traumatology [25]. As the majority of clavicle fracture patients are under 40 years of age [18, 26], the important outcomes of clavicle fracture treatment are absence from work and cost-effectiveness. Two studies have previously reported that operatively treated patients may return to work or sports earlier compared with non-operatively treated patients [14, 27•], but two studies have shown no difference [18, 28••]. However, in the study of Pearson et al., they reported that surgical intervention is relatively expensive, if the duration of functional benefit is assumed to persist for only 1 year [26].

The aim of this systematic review is to investigate the cost-effectiveness of operative and non-operative clavicle fracture treatment. We have three objectives regarding the cost-effectiveness in this study: (1) Which treatment is more expensive in clavicle fracture treatment—the non-operative or operative method? (2) Which costs have been included in the published literature? (3) Which methods have been used to calculate the cost-effectiveness in clavicle fracture treatment?

## Methods

### Overview and Eligibility Criteria for Review

A literature review was performed based on the Preferred Reporting Items for Systematic Reviews and Meta-analyses (PRISMA) [29]. The review protocol for this study was created by the authors and is available as a supplementary file.

In the literature review, we included publications concerning the costs or cost-effectiveness of operative and non-operative treatment of acute fractures of the clavicle. The exclusion criteria for the report of data were the following: (1) duplication, (2) case reports, (3) only the cost-effectiveness results of operative and non-operative treatment were reported, (4) adolescent studies, and (5) non-English publications.

As the treatment methods and recovery are similar in all three fracture locations (midshaft, lateral, or medial), we decided to include all clavicle fracture publications that reported the cost-effectiveness results of operative or non-operative treatment, and hereafter we use the term “clavicle fracture.”

### Search Methods

For the literature search, the PubMed, Ovid, Scopus, Web of Science, and EBSCO host databases were used. All published articles were retrieved without a search constraint on March 9, 2020. The following keywords combined with Medical Subject Headings (MeSH) terms were used in the search: “economic OR cost OR costs OR cost savings OR effective OR cost-effective OR cost effectiveness OR cost effectiveness” and “clavicle OR clavicular OR collar bone.”

**Fig. 1:**
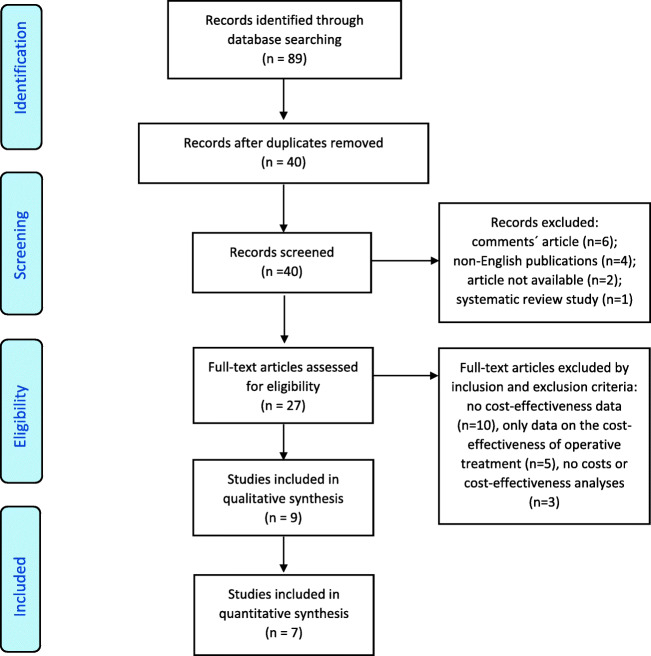
Flow diagram showing flow of studies retrieved for systematic review of operative versus non-operative treatment cost-effectiveness studies for clavicular fractures

The two authors (KG and RL) independently reviewed all the titles and appropriate abstracts manually. All unsuitable articles were excluded using the predefined set of exclusion criteria. A manual search was performed for all references of suitable studies by reviewing titles and appropriate abstracts. The included studies were reviewed and added to the final list using the set inclusion criteria. Disagreements in data extraction were resolved by discussion and consensus between the authors (KG, RL, LAP, and MVM) (Fig. 1).

### Quality Assessment

The Quality of Health Economic Studies (QHES) instrument was used in the assessment of the quality of the included studies. This instrument is a validated questionnaire that includes 16 questions with weighted binary responses that range from 1 to 9 points [30]. The QHES instrument is health economic-specific. Scores for the QHES instrument range from 0 to 100, and there is no accepted cut-off value to define the high quality of a study. Since the introduction and application of the QHES instrument, the reported mean and median scores have been between 80 and 90. Therefore, the authors considered a QHES score of greater than 85 as an indicator of high quality [31–34].

The level of evidence was stated for each article as reported by the original journal.

### Study Data

Two independent investigators (KG and RL) collected information from the included publications using a standardized data collection form. Details of the included study type, year, the main characteristics of patients, and the results concerning the cost-effectiveness of treatment were extracted by one investigator and verified by the second investigator.

All costs in this study are presented in US dollar (USD). The latest study data were based on 2020 prices. The prices in the studies were discounted at an annual discount rate of 3% to the year 2020, due to general inflation of prices, in accordance with the recommendations of the Panel on Cost-Effectiveness in Health and Medicine [35•].

To present the mean cost for treatment per person, the average cost of non-operative and operative treatment was calculated by averaging each study with equal weight, which is in deviation to the conventional weighting according to the sample size of the study. After that, the average cost of non-operative and operative treatment methods was calculated based on the results of all the included studies. The same method of calculation was used to present mean absence from work.

A meta-analysis was not performed because of the heterogeneity of the patients and methods for calculating the cost-effectiveness.

## Results

### Study Selection

The electronic searches produced 2880 articles. The title and abstract of all articles were screened manually. All publications on clavicle fracture treatment and cost-effectiveness were then selected (*n* = 89), and 40 articles were extracted (49 of 89 were duplicates). The study selection process is presented in Fig. 1. All references of relevant studies in full-text review (*n* = 23) were controlled for suitable related citations as an additional screening through other sources, and 23 articles were identified. All these 23 articles were duplicates of the primary screening results.

In the first phase, 27 of the 40 articles, and in second, 9 [18, 26, 28••, 36, 37•, 38, 39•, 40, 41] of the 27 articles were selected using inclusion and exclusion criteria (Fig. 1).

In a discussion on the final included publications, the authors noticed that two publications [26, 41] contained a part of same data and two exactly the same data [18, 38] that would have led to bias in the analysis process. In the study by Walton and colleagues, the data included data from 4 RCT studies [8, 13, 42, 43]. The Pearson et al. publication was based on one RCT study data [13] that was also included in Walton et al.’s study. Nicholson et al.’s analysis was performed based on Robinson et al.’s RCT study, which was more precise in cost-effective analysis point of view. After thorough discussion, the author group decided to exclude the Walton et al. and Robinson et al. studies.

In six of the seven included publications, the cost-effectiveness of treatment for midshaft clavicle fractures had been studied [26, 36, 37•, 38, 39•, 40], and the seventh one studied clavicle fractures with unspecified fracture location [28^••^]. All seven publications were included [26, 28••, 36, 37•,^38^, ^39^•, 40] (Table 1). The characteristics of the patients in the included studies are summarized in Table 2.

**Table 1:** Included publications

Article author	Year	Country	Study type	QHES	Level of evidence
Althausen et al.	2013	USA	Retrospective case-control design	45	3
Pearson et al.	2010	Canada	Formal cost-effectiveness analysis based on a prospective data	96	1
Shields et al.	2016	USA	Retrospective database study	57	2
Liu et al.	2019	USA	Formal cost-effectiveness analysis based on a prospective and retrospective data	100	2
Nicholson et al.	2019	UK	Formal cost-analysis based on RCT study	73	
Sørensen et al.	2019	Denmark	Decision analytical modeling based on RCT study	75	1
Herteleer et al.	2020	Belgium	Retrospective study	62	3

**Table 2:** The characteristics of the patients in the included studies

Characteristics	Althausen et al.	Pearson et al.	Shields et al.	Liu et al.	Nicholson et al.	Sørensen et al.	Herteleer et al.
Patients in study (*n*)	149	111	169	N	178	133	345
Sex							
Male	112	87	N	N	158	N	280
Female	37	24	N	N	20	N	65
Mean age (y)							
Operative	40	34	N	35	32	N	36
Non-operative	42	34	N	35	33	N	23
Tobacco use (%)							
Operative	12	31	N	N	23	N	28
Non-operative	8	33	N	N	22	N	N
Treatment (*n*)							
Non-operative	83	49	135	N	92	65	108
Operative	66	62	34	100%	86	68	237
Plate fixation	66	62	N	79%	86	N	N
Follow-up in year	3	1	N	5	1	N	N
Non-union (%)							
Operative	0	3	N	4	1	N	N
Non-operative	5	14	N	N	17	19	N
Infection (%)	0	5	N	N	2 s.i	2	2
Need for further surgery (%)							
Operative	5	N	N	N	19	N	21
Non-operative	0	N	N	N	18	N	N
Hardware irritation required removal (%)	N	8	12	N	N	12	38

*n*, number of patients; *N*, not presented or calculated; *s.i*, superficial infection; *y*, year

### Cost-Effectiveness

Five of the 7 studies found that routine operative treatment for displaced clavicle fracture was more expensive than non-operative treatment [26, 28••, 38, 39•, 40]. In two studies, despite that operative treatment is more expensive, it might be more cost-effective in selected population, for example, in population requiring better earlier functional outcome [26, 39•]. In the works of Liu et al. and Sorensen et al., no actual amounts of patients (including ratio of operative vs non-operative treatment) were presented [37•, 39•]. Therefore, these studies’ data were not used in calculations of overall mean costs for operative and non-operative treatment. The mean overall cost per person in discounted prices were 10,230 USD for operative and 7923 USD for non-operative treatment (Table 3). In 2 of 3 studies, return to work was presented; operative treatment was also found to be more expensive than non-operative treatment [23, 26] (Tables 3 and 4). In studies where absence from work was calculated, the mean cost per person in discounted prices for operative and non-operative treatment were 18,589 USD and 16,691 USD, respectively (Tables 3 and 4).

**Table 3:** Overview of costs for clavicle fracture treatment in USD*

	Costs per person (USD)		Hospital care costs (USD)		Original currency
	Operative	Non-operative	Operative	Non-operative	
Althausen et al.	15,960	22,222	10,479	4541	USD
Pearson et al.	21,387	7321	7175	249	USD
Shields et al.	32,793	9416			USD
Nicholson et al	7295	1785			GBP
Herteleer et al.	3543	570			EUR
Average	10,230	7923	8879	2948	
Average**	18,589	16,691			

*The prices were discounted at an annual discount rate of 3% to the year 2020. GBP are calculated to USD (currency 1:1.15) and EUR to USD (currency 1:1.31)

**Average of articles (Althausen et al., Pearson et al) where absence of work was calculated

*GBP*, United Kingdom Pound; *USD*, United States dollar; *EUR*, the official currency of the European Union

**Table 4:** Patients’ absence from work (in days)

Characteristics	Althausen et al.	Shields et al.	Nicholson et al.
Patient missed from work			
Operative	8	196	22
Non-operative	35	69	24
Family member missed from work			
Operative	3	N	N
Non-operative	7		
When released to full duty			
Operative	36	N	N
Non-operative	61		

*N*, not presented or calculated

### Absence from Work

All studies have taken into account patients’ absence from work and included it in analysis. Information concerning patients’ absence from work in days was reported in 3 of 7 publications. The duration of absence from work varied between 8–195 and 35–69 days, and the means were 49 and 47 days for operative and non-operative treatment, respectively.

All publications used different methods for extracting information on absence from work. Althausen et al. used a financial questionnaire that was mailed to patients (available on the original journal’s website) [36]. The questionnaire had both a clinical and a financial component that included return-to-work date. Nicholson et al. did not present the method they used to extract the information on absence from work [38]. Shields et al. extracted the information from the Workers’ Compensation national database [28••].

### Mode of Economic Analysis

Decision modeling is used in 3 of 7 studies [26, 37•, 39•]. Quality-adjusted life-years (QALYs) is used in 3 of 7 studies [26, 38, 39•]. Different variables are analyzed in 3 of 7 studies [28••, 36, 40].

In three studies, costs are based on local health care financing system and costs of hospital care [38, 39•, 40]. Two studies’ costs were based on the national average Medicare reimbursements [26, 37•], and one based the costs on the Workers’ Compensation national database [28••]. One study’s cost was based on hospital financial records and financial questionnaires, which were mailed to patients [36].

### Quality Assessment

The methodological quality and the level of evidence of the 7 studies are summarized in Table 1. The mean QHES result was 72.6. The minimum and maximum scores were 45 and 100, respectively. Five of 7 studies’ level of evidence were reported by the original journal. As Pearson et al.’s study level of evidence was not reported and this cost-effectiveness work was part of the RCT study, the level of evidence was set by the original study [13, 26]. One study level of evidence was not reported [38]. The lowest level of evidence was III.

## Discussion

The main finding of our systematic review was that the routine operative treatment of displaced clavicle fracture is more expensive than the non-operative treatment. The mean costs per person were 10,230 USD for operative and 7923 USD for non-operative treatment. This finding is similar to a study by Walton and colleagues; they concluded that the non-operative treatment of midshaft clavicle fractures is less costly than the operative treatment [41]. Furthermore, the study of Robinson et al. did not support routine primary open reduction and plate fixation for the treatment of displaced midshaft clavicular fractures [18]. However, the heterogeneity of cost-effectiveness calculations and of absence from work leads to uncertainty of further conclusions from the data. In the end, the length of the absence from work after clavicle fracture is influenced by physical demand of the work among other personal-level factors. Thus, it is likely to exist heterogeneity in cost-effectiveness of operative and non-operative between subpopulations of clavicle fracture patients, even though routine operative fixation does not seem to be cost-effective based on the current knowledge.

In high-quality RCTs, it has been shown that 1- and 2-year functional differences between the operative and non-operative treatment of clavicle fracture are minimal or do not exist [9, 44, 45]. However, the results are contradicting and partially confusing. In one hand, the problem is heterogeneity of the reported outcomes, and in other hand, patient-reported outcome measures (PROMs) have their own problems as variables. There is a lack of context-specific (for example age- and fracture-specific) minimal clinically important differences [46••], and therefore, the interpretation of the results may be challenging [47••]. In particular, the possibility of ceiling effect in utilized PROMs among young and physically active population may diminish small albeit perhaps important differences between operative and non-operative patients [48–50].

Operations are common after both treatments [9]. It has been suggested that in short-term follow-up (less than 1 year), operative treatment may be associated with better functional outcome [12, 44]. Interestingly, better short-term functional outcome does not necessary equate with a quicker return to work [8, 9]. Absence from work is a crucial factor in calculating cost-effectiveness in the working aged population. In the present review, the overall mean difference in absence from work for operative and non-operative treatment groups was 2 days (49 and 47). It must be remembered, however, that occupational demand, self-reported disability/pain [51], injury severity, and presence of injury compensation [52] have also been shown to influence return to work. Moreover, the mean duration of absence from work varied greatly among the studies included in this review (Table 4).

The relevant costs and health consequences that are included in a cost-effectiveness trial may differ based on the study question. A study might use a patient, hospital, insurer, or society perspective. For example, in the original publications included in this review, absence from work was measured in at least three different ways. Althausen et al. used a questionnaire, which may lack accuracy and include recall bias [36]; Robinson et al., on the other hand, did not present the method used to acquire the information on absence from work [18]; and finally, Liu et al. averaged the mean absence from work from previous studies [37^•^]. Shields at al. extracted the information from the Workers’ Compensation national database which, in our opinion, represents the most valid method for gathering data on absence from work [28^••^], in cases were all injuries and patients are added to the database.

There is no universal agreement on issues that should be taken into account when evaluating the cost-effectiveness of health care interventions. In the UK, the National Institute for Health and Care Excellence (NICE) has suggested that the costs incurred from absence from work should not be taken into account in the cost-effectiveness evaluations of health interventions [53]. The primary argument for this view is the belief that this kind of health intervention evaluation could lead to unethical and unequal prioritization intervention decisions being made, e.g., between employed and unemployed people or between men and women [54]. Other opinions also exist. A recent study assessed European-wide standpoints from health economic experts about the conducting of economic evaluations [55••]. The study found agreement among experts on using the value of productivity loss as part of the opportunity costs of the investigated health issue, it but found some disagreement on the actual techniques to be used in measuring the productivity loss.

The QHES quality scores for the included studies varied from 45 [36] to 100 [37•]. A QHES score of greater than 85 can be seen as an indicator of high quality. The study by Shield et al. achieved low QHES score [28••]. In addition, the study did not report the anatomical localization of the clavicle fractures (medial, midshaft, lateral); no information was reported about patient-reported outcomes; there was an absence of details about the non-operative treatment regimens or surgical techniques. If the results would have been analyzed without Shields’ study, the operatively treated patients’ return to work would have been faster than that of non-operatively treated patients. However, reports of absence from work are controversial, and this very important aspect of cost-effectiveness is still unclear. In addition, we used QHES to measure the quality of original publications.

To our knowledge, this literature review is the first that has been published in this field. The strengths of this review are the use of PRISMA guidelines, the reporting of the results without a search constraint, and the methodological quality assessment for this topic. Our study had limitations. Publications in this review are heterogenic as discussed previously. Analyses are performed using different methods, and in one study, the actual fracture type is unknown. Health financial systems in different countries are not similar, which jeopardize the homogenic comparison.

## Conclusions

In conclusion, the main finding of our systematic review was that the routine operative treatment of clavicle fracture is more expensive than the non-operative treatment. It might be more cost-effective in selected patients, e.g., patients who require better earlier functional outcome. The included direct and indirect costs related to interventions varied profoundly within the studies. Different ways are used to calculate the cost-effectiveness of operative and non-operative treatment. Absence from work was included in three studies, but due to large heterogeneity, the validity of the result in this aspect may be questioned.

## Electronic supplementary material

ESM1 (PDF 76.4 kb)

ESM2 (DOC 63.5 kb)

## Data Availability

The review protocol for this study is available as a supplementary file.

## Abbreviations

RCT: randomized controlled trial
QHES: Quality of Health Economic Studies
USD: United States dollar
PRISMA: Preferred Reporting Items for Systematic Reviews and Meta-analyses
NICE: National Institute for Health and Care Excellence
