# Supplementary material for: Cost-Effectiveness of Operative Versus Non-Operative Treatment for Clavicle Fracture: a Systematic Literature Review
Source: Curr Rev Musculoskelet Med. 2020 May 8;13(4):391–9. doi: 10.1007/s12178-020-09640-0 (PMC7340703; doi:10.1007/s12178-020-09640-0)
Supplement: Supplementary file 1 — (PDF 76.4 kb) [file 12178_2020_9640_MOESM1_ESM.pdf]

Title: **Operative and nonoperative treatmen of clavicle fractures: literature review of cost-effectiveness**

Date: **3/10/2020**

Author: Gilber Kask, MD, MBA
Lauri Raittio B.M & B.Sc. Econ

Search words: (economic OR cost OR costs OR cost savings OR effective OR cost-effective OR cost effectiveness OR cost effectiveness) and (clavicle OR clavicular OR collarbone)

Nr:	Author Ovid search:	Title	Year	Source	Journal
				**Results: 513**	
1	Sørensen et al.	Operative Treatment of Displaced Midshaft Clavicular Fractures Is Not Cost-Effective	2020	Ovid	J Shoulder Elbow Surg
2	Nicolson et al.	Routine Fixation of Displaced Midshaft Clavicle Fractures Is Not Cost-Effective: A Cost Analysis From a Randomized Controlled Trial	2019	Ovid	Bone Joint J
3	Benson et al.	My Clavicle Fracture Definitely Warrants Surgery-Or Not So Much?: Commentary on an article by Jane Liu, MD, et al.: "Cost-Effectiveness of Operative Versus Nonoperative Treatment of Displaced Midshaft Clavicle Fractures. A Decision Analysis"	2019	Ovid	Journal of Bone & Joint Surgery - American Volume
4	Liu et al.	Cost-Effectiveness of Operative Versus Nonoperative Treatment of Displaced Midshaft Clavicle Fractures: A Decision Analysis.	2019	Ovid	Journal of Bone & Joint Surgery - American Volume
5	Tamaoki et al.	Treatment of Displaced Midshaft Clavicle Fractures: Figure-of-Eight Harness Versus Anterior Plate Osteosynthesis: A Randomized Controlled Trial	2017	Ovid	Journal of Bone & Joint Surgery - American Volume
6	Tutuhatunewa et al.	Clinical outcomes and predictors of patient satisfaction in displaced midshaft clavicle fractures in adults: Results from a retrospective multicentre study.	2017	Ovid	Injury
7	Tiren et al.	Superior clavicle plate with lateral extension for displaced lateral clavicle fractures: a prospective study.	2013	Ovid	*Journal of Orthopaedics & Traumatology.*
8	Lee et al.	Comparison of the efficacy of hook plate versus tension band wire in the treatment of unstable fractures of the distal clavicle.	2009	Ovid	*International Orthopaedics.*
9	Lädermann et al.	Functional recovery following early mobilization after middle third clavicle osteosynthesis for acute fractures or nonunion: A case-control study.	2017	Ovid	Orthop Traumatol Surg Res.
10	Hanselman et al.	Operative Cost Comparison: Plating Versus Intramedullary Fixation for Clavicle Fractures.	2016	Ovid	Orthopedics.
11	Shields et al.	Patient factors influencing return to work and cumulative financial claims after clavicle fractures in workers' compensation cases.	2016	Ovid	J Shoulder Elbow Surg.
12	Walton et al.	A cost analysis of internal fixation versus nonoperative treatment in adult midshaft clavicle fractures using multiple randomized controlled trials.	2015	Ovid	J Orthop Trauma.
13	Melean et al.	Surgical treatment of displaced middle-third clavicular fractures: a prospective, randomized trial in a working compensation population.	2015	Ovid	J Shoulder Elbow Surg.
14	Althausen et al.	Clinical and financial comparison of operative and nonoperative treatment of displaced clavicle fractures.	2013	Ovid	J Shoulder Elbow Surg.
15	Robinson et al.	Open reduction and plate fixation versus nonoperative treatment for displaced midshaft clavicular fractures: a multicenter, randomized, controlled trial	2013	Ovid	J Bone Joint Surg Am.
16	Lai et al.	Comparison of dynamic and locked compression plates for treating midshaft clavicle fractures.	2012	Ovid	Orthopedics.
17	Pearson et al.	Is surgery for displaced, midshaft clavicle fractures in adults cost-effective? Results based on a multicenter randomized, controlled trial.	2010	Ovid	J Orthop Trauma.

Nr:	Author SCOPUS search:	Title	Year	Source	Journal
				**Results: 509**	
18	Sørensen et al.	Operative Treatment of Displaced Midshaft Clavicular Fractures Is Not Cost-Effective	2020	Scopus	J Shoulder Elbow Surg
19	Fox et al.	Neer Type-II Distal Clavicle Fractures: A Cost-Effectiveness Analysis of Fixation Techniques	2020	Scopus	J Bone Joint Surg Am
20	Herteleer et al.	Healthcare Utilization and Related Cost of Midshaft Clavicle Fracture Treatment in Belgium	2020	Scopus	Eur J Trauma Emerg Surg
21	Nicolson et al.	Routine Fixation of Displaced Midshaft Clavicle Fractures Is Not Cost-Effective: A Cost Analysis From a Randomized Controlled Trial	2019	Scopus	Bone Joint J

22	Benson et al.	My Clavicle Fracture Definitely Warrants Surgery-Or Not So Much?: Commentary on an article by Jane Liu, MD, et al.: "Cost-Effectiveness of Operative Versus Nonoperative Treatment of Displaced Midshaft Clavicle Fractures. A Decision Analysis"	2019	Scopus	Journal of Bone & Joint Surgery - American Volume
23	Liu et al.	Cost-Effectiveness of Operative Versus Nonoperative Treatment of Displaced Midshaft Clavicle Fractures: A Decision Analysis.	2019	Scopus	Journal of Bone & Joint Surgery - American Volume
24	Tamaoki et al.	Treatment of Displaced Midshaft Clavicle Fractures: Figure-of-Eight Harness Versus Anterior Plate Osteosynthesis: A Randomized Controlled Trial	2017	Scopus	Journal of Bone & Joint Surgery - American Volume
25	Walton et al.	A cost analysis of internal fixation versus nonoperative treatment in adult midshaft clavicle fractures using multiple randomized controlled trials	2015	Scopus	J Orthop Trauma.
26	Melean et al.	Surgical treatment of displaced middle-third clavicular fractures: A prospective, randomized trial in a working compensation population	2015	Scopus	Journal of Shoulder and Elbow Surgery
27	Douraiswami et al.	Open reduction and plating for displaced mid third clavicle fractures - A prospective study	2013	Scopus	Journal of Clinical Orthopaedics and Trauma
28	Robinson et al.	Open reduction and plate fixation versus nonoperative treatment for displaced midshaft clavicular fractures: a multicenter, randomized, controlled trial	2013	Scopus	J Bone Joint Surg Am.
29	Böhme et al.	Current treatment concepts for mid-shaft fractures of the clavicle - Results of a prospective multicentre study | [Aktuelle Behandlungskonzepte der Klavikulaschaftfraktur - Ergebnisse einer prospektiven Multicenterstudie]	2011	Scopus	Zeitschrift fur Orthopadie und Unfallchirurgie
30	Lädermann et al.	Functional recovery following early mobilization after middle third clavicle osteosynthesis for acute fractures or nonunion: A case-control study	2017	Scopus	Orthopaedics and Traumatology: Surgery and Research
31	Hanselman et al.	Operative cost comparison: Plating versus intramedullary fixation for clavicle fractures	2016	Scopus	Orthopedics
32	Shields et al.	Patient factors influencing return to work and cumulative financial claims after clavicle fractures in workers' compensation cases	2016	Scopus	Journal of Shoulder and Elbow Surgery
33	Althausen et al.	Clinical and financial comparison of operative and nonoperative treatment of displaced clavicle fractures	2013	Scopus	Journal of Shoulder and Elbow Surgery
34	Pearson et al.	Is surgery for displaced, midshaft clavicle fractures in adults cost-effective? Results based on a multicenter randomized, controlled trial	2010	Scopus	Journal of Orthopaedic Trauma

**Nr:**	**Author Web of science search:**	**Title**	**Year**	**Source**	**Journal**
				**Results: 421**	
35	Sørensen et al.	Operative Treatment of Displaced Midshaft Clavicular Fractures Is Not Cost-Effective	2020	Web of science	J Shoulder Elbow Surg
36	Benson et al.	My Clavicle Fracture Definitely Warrants Surgery-Or Not So Much?: Commentary on an article by Jane Liu, MD, et al.: "Cost-Effectiveness of Operative Versus Nonoperative Treatment of Displaced Midshaft Clavicle Fractures. A Decision Analysis"	2019	Web of science	Journal of Bone & Joint Surgery - American Volume
37	Liu et al.	Cost-Effectiveness of Operative Versus Nonoperative Treatment of Displaced Midshaft Clavicle Fractures: A Decision Analysis.	2019	Web of science	Journal of Bone & Joint Surgery - American Volume
38	Tamaoki et al.	Treatment of Displaced Midshaft Clavicle Fractures: Figure-of-Eight Harness Versus Anterior Plate Osteosynthesis: A Randomized Controlled Trial	2017	Web of science	Journal of Bone & Joint Surgery - American Volume
39	Serrano et al.	Anterior-Inferior Plating Results in Fewer Secondary Interventions Compared to Superior Plating for Acute Displaced Midshaft Clavicle Fractures.	2017	Web of science	Journal of Orthopaedic Trauma,
40	Melean et al.	Surgical treatment of displaced middle-third clavicular fractures: A prospective, randomized trial in a working compensation population	2015	Web of science	Journal of Shoulder and Elbow Surgery
41	Hanselman et al.	Operative Cost Comparison: Plating Versus Intramedullary Fixation for Clavicle Fractures	2016	Web of Science	Orthopedics
42	Shields et al.	Patient factors influencing return to work and cumulative financial claims after clavicle fractures in workers' compensation cases	2016	Web of Science	JOURNAL OF SHOULDER AND ELBOW SURGERY
43	Bliss et al.	Does Insurance Status Affect the Management of Acute Clavicle Fractures?	2016	Web of Science	JOURNAL OF ORTHOPAEDIC TRAUMA
44	Walton et al.	A Cost Analysis of Internal Fixation Versus Nonoperative Treatment in Adult Midshaft Clavicle Fractures Using Multiple Randomized Controlled Trials	2015	Web of Science	JOURNAL OF ORTHOPAEDIC TRAUMA
45	Frischknecht et al.	Internal fixation of the midshaft clavicle with 3.5mm reconstruction LC-Plate - a safe, adaptable and cost effective option	2014	Web of Science	BRITISH JOURNAL OF SURGERY

46	Robinson et al.	Open Reduction and Plate Fixation Versus Nonoperative Treatment for Displaced Midshaft Clavicular Fractures A Multicenter, Randomized, Controlled Trial	2013	Web of Science	JOURNAL OF BONE AND JOINT SURGERY-AMERICAN
47	Pearson et al.	Is Surgery for Displaced, Midshaft Clavicle Fractures in Adults Cost-Effective? Results Based on a Multicenter Randomized, Controlled Trial	2010	Web of Science	. JOURNAL OF ORTHOPAEDIC TRAUMA

**Nr:**	**Author EBSCO host search:**	**Title**	**Year**	**Source**	**Journal**
				**Results: 906**	
48	Fox et al.	Neer Type-II Distal Clavicle Fractures: A Cost-Effectiveness Analysis of Fixation Techniques	2020	EBSCO host	J Bone Joint Surg Am
49	Morales et al.	Potential Economic Benefits of Limited Clinical and Radiographic Follow-up After Plate Fixation of Midshaft Clavicle Fractures	2019	EBSCO host	Journal of the American Academy of Orthopaedic Surgeons
50	Benson et al.	My Clavicle Fracture Definitely Warrants Surgery-Or Not So Much?: Commentary on an article by Jane Liu, MD, et al.: "Cost-Effectiveness of Operative Versus Nonoperative Treatment of Displaced Midshaft Clavicle Fractures. A Decision Analysis"	2019	EBSCO host	Journal of Bone & Joint Surgery - American Volume
51	Liu et al.	Cost-Effectiveness of Operative Versus Nonoperative Treatment of Displaced Midshaft Clavicle Fractures: A Decision Analysis.	2019	EBSCO host	Journal of Bone & Joint Surgery - American Volume
52	Tamaoki et al.	Treatment of Displaced Midshaft Clavicle Fractures: Figure-of-Eight Harness Versus Anterior Plate Osteosynthesis: A Randomized Controlled Trial	2017	EBSCO host	Journal of Bone & Joint Surgery - American Volume
53	Serrano et al.	Anterior-Inferior Plating Results in Fewer Secondary Interventions Compared to Superior Plating for Acute Displaced Midshaft Clavicle Fractures.	2017	EBSCO host	Journal of Orthopaedic Trauma,
54	Hanselman et al.	Operative Cost Comparison: Plating Versus Intramedullary Fixation for Clavicle Fractures.	2016	EBSCO host	Orthopedics
55	Mcphillamy et al.	The Clinical and Economic Impact of Generic Locking Plate Utilization at a Level II Trauma Center.	2016	EBSCO host	Journal Of Orthopaedic Trauma
56	Shields et al.	Patient factors influencing return to work and cumulative financial claims after clavicle fractures in workers' compensation cases.	2016	EBSCO host	Journal Of Shoulder And Elbow Surgery
57	Melean et al.	Surgical treatment of displaced middle-third clavicular fractures: A prospective, randomized trial in a working compensation population	2015	EBSCO host	Journal of Shoulder and Elbow Surgery
58	Walton et al.	A cost analysis of internal fixation versus nonoperative treatment in adult midshaft clavicle fractures using multiple randomized controlled trials.	2015	EBSCO host	Journal of Orthopaedic Trauma (J ORTHOP TRAUMA)
59	Robinson et al.	Open reduction and plate fixation versus nonoperative treatment for displaced midshaft clavicular fractures: a multicenter, randomized, controlled trial.	2013	EBSCO host	The Journal Of Bone And Joint Surgery.
60	Althausen et al.	Clinical and financial comparison of operative and nonoperative treatment of displaced clavicle fractures.	2013	EBSCO host	Journal Of Shoulder And Elbow Surgery
61	Böhme et al.	Current treatment concepts for mid-shaft fractures of the clavicle - Results of a prospective multicentre study | [Aktuelle Behandlungskonzepte der Klavikulaschaftfraktur - Ergebnisse einer prospektiven Multicenterstudie]	2011	EBSCO host	Zeitschrift fur Orthopadie und Unfallchirurgie
62	Pearson et al.	Is surgery for displaced, midshaft clavicle fractures in adults cost-effective? Results based on a multicenter randomized, controlled trial.	2010	EBSCO host	Journal of Orthopaedic Trauma (J ORTHOP TRAUMA)

**Nr:**	**Author PubMed search:**	**Title**	**Year**	**Source**	**Journal**
				**Results: 531**	
63	Sørensen et al.	Operative Treatment of Displaced Midshaft Clavicular Fractures Is Not Cost-Effective	2020	Pub Med	J Shoulder Elbow Surg
64	Fox et al.	Neer Type-II Distal Clavicle Fractures: A Cost-Effectiveness Analysis of Fixation Techniques	2020	Pub Med	J Bone Joint Surg Am
65	Herteleer et al.	Healthcare Utilization and Related Cost of Midshaft Clavicle Fracture Treatment in Belgium	2020	Pub Med	Eur J Trauma Emerg Surg
66	Putnamn et al.	Surgical Treatment, Complications, and Reimbursement Among Patients With Clavicle Fracture and Acromioclavicular Dislocations: A US Retrospective Claims Database Analysis	2019	Pub Med	J Med Econ
67	Nicolson et al.	Routine Fixation of Displaced Midshaft Clavicle Fractures Is Not Cost-Effective: A Cost Analysis From a Randomized Controlled Trial	2019	Pub Med	Bone Joint J

68	Benson et al.	My Clavicle Fracture Definitely Warrants Surgery-Or Not So Much?: Commentary on an article by Jane Liu, MD, et al.: "Cost-Effectiveness of Operative Versus Nonoperative Treatment of Displaced Midshaft Clavicle Fractures. A Decision Analysis"	2019	Pub Med	Journal of Bone & Joint Surgery - American Volume
69	Lui et al.	Cost-Effectiveness of Operative Versus Nonoperative Treatment of Displaced Midshaft Clavicle Fractures: A Decision Analysis.	2019	Pub Med	Journal of Bone & Joint Surgery - American Volume
70	Tamaoki et al.	Treatment of Displaced Midshaft Clavicle Fractures: Figure-of-Eight Harness Versus Anterior Plate Osteosynthesis: A Randomized Controlled Trial	2017	Pub Med	Journal of Bone & Joint Surgery - American Volume
71	Althausen et al.	Clinical and financial comparison of operative and nonoperative treatment of displaced clavicle fractures.	2013	Pub Med	J Shoulder Elbow Surg.
72	Pearson et al.	Is surgery for displaced, midshaft clavicle fractures in adults cost-effective? Results based on a multicenter randomized, controlled trial.	2010	Pub Med	J Orthop Trauma.
73	Lai et al.	Comparison of dynamic and locked compression plates for treating midshaft clavicle fractures.	2012	Pub Med	Orthopedics.
74	Meijer et al.	In response.	2015	Pub Med	J Orthop Trauma.
75	Qamar et al.	Double semi-tubular plating of clavicle using a piggyback technique--an alternative way of treating clavicle mid-shaft fractures in young patients.	2011	Pub Med	Acta Orthop Belg.
76	Walton et al.	A cost analysis of internal fixation versus nonoperative treatment in adult midshaft clavicle fractures using multiple randomized controlled trials.	2015	Pub Med	J Orthop Trauma.
77	Robinson et al.	Open reduction and plate fixation versus nonoperative treatment for displaced midshaft clavicular fractures: a multicenter, randomized, controlled trial	2013	Pub Med	J Bone Joint Surg Am.
78	Garg et al.	Displaced middle-third fractures of the clavicle-operative management.	2011	Pub Med	J Indian Med Assoc
79	Li et al.	[Surgical and nonsurgical treatment of clavicular midpiece fracture in adults: a case-control study].	2012	Pub Med	Zhongguo Gu Shang.
80	Rehn et al.	Operative versus nonoperative treatment of displaced midshaft clavicle fractures in adults: a systematic review	2014	Pub Med	Eur J Orthop Surg Traumatol.
81	Melean et al.	Surgical treatment of displaced middle-third clavicular fractures: a prospective, randomized trial in a working compensation population.	2015	Pub Med	J Shoulder Elbow Surg.
82	Hanselman et al.	Operative Cost Comparison: Plating Versus Intramedullary Fixation for Clavicle Fractures.	2016	Pub Med	Orthopedics.
83	Durak et al.	[Results of conservative treatment of midclavicular fractures].	2002	Pub Med	Ulus Travma Derg.
84	Shields et al.	Patient factors influencing return to work and cumulative financial claims after clavicle fractures in workers' compensation cases.	2016	Pub Med	J Shoulder Elbow Surg.
85	Zhang et al.	[Minimally invasive treatment for fresh acromioclavicular dislocation and the distal clavicle fracture].	2011	Pub Med	Zhongguo Gu Shang.
86	Czajka et al.	Symptomatic Implant Removal Following Dual Mini-Fragment Plating for Clavicular Shaft Fractures.	2017	Pub Med	J Orthop Trauma.
87	Mackey et al.	Effects of antiresorptive treatment on nonvertebral fracture outcomes.	2011	Pub Med	J Bone Miner Res.
88	Lu et al.	Superior versus anteroinferior plating of displaced midshaft clavicular fracture in patients older than 60 years.	2017	Pub Med	J Int Med Res.
89	Lädermann et al.	Functional recovery following early mobilization after middle third clavicle osteosynthesis for acute fractures or nonunion: A case-control study.	2017	Pub Med	Orthop Traumatol Surg Res.
